# Percutaneous radial access is safe and effective for subclavian branch treatment during zone 2 endovascular aortic repair with thoracic branch endoprosthesis

**DOI:** 10.1016/j.jvscit.2025.101922

**Published:** 2025-07-10

**Authors:** Matthew Schneck, Kathryn Dilosa, Matthew Mell, Rachael Callcut, Steven Maximus

**Affiliations:** a Division of Vascular Surgery, University of California Davis, Sacramento, CA; b Division of Trauma and Acute Care Surgery, University of California Davis, Sacramento, CA; c Division of Vascular Surgery, Baylor College of Medicine, Houston, TX

**Keywords:** Endovascular aneurysm repair, Aorta, Thoracic, Radial artery, Subclavian artery, Blood vessel prosthesis

## Abstract

The thoracic branch endoprosthesis (TBE, W. L. Gore & Associates) provides an off-the-shelf endovascular solution for zone 2 aortic pathology that maintains perfusion to the arm. This study evaluates safety and efficacy of percutaneous radial access for subclavian branch treatment during TBE repair. A single-institution retrospective review of consecutive patients who underwent zone 2 aortic repair with TBE between 2022 and 2024 was performed. Treatment indications included acute complicated aortic dissection, aortic dissection with aneurysmal degeneration, penetrating aortic ulcer, blunt thoracic aortic injury, and de novo aneurysmal disease. Sixty-three consecutive patients (66.7% male; mean age, 61.0 ± 16.5 years) underwent zone 2 repair with a TBE device, 29 (46.0%) for aneurysmal disease, 15 (23.8%) for blunt thoracic aortic injury, 17 (27.0%) for dissection, and 2 (3.2%) for penetrating aortic ulcer. Twenty-one (33.3%) were urgent or emergent cases performed within 24 hours of presentation. Side branch treatment was performed through percutaneous left radial access in 60 patients (95.2%). Radial access was technically successful in 100% of attempts without the need for more proximal percutaneous or open upper extremity access. One (1.7%) asymptomatic radial artery occlusion was detected postoperatively, and did not require intervention. Postoperative imaging was obtained before discharge in 58 patients (92.1%), demonstrating 100% subclavian side branch patency. These results demonstrate that left radial access is safe and effective for subclavian branch treatment during thoracic branch endoprosthesis for management of zone 2 aortic pathology.

As endovascular technology continues to progress, treatment paradigms for thoracic aortic pathology perpetually shift. Once the mainstay of management, open reconstruction has been supplanted by thoracic endovascular aortic repair (TEVAR) for repair in descending thoracic aortic disease. When pathology encroaches on zone 2, coverage of the left subclavian artery (LSA) to achieve adequate proximal seal is required. The need for LSA coverage has been reported in 10% to 50% of cases, depending on practice and referral patterns.^1^ When revascularization of the LSA is indicated per Society for Vascular Surgery guidelines, it is performed traditionally with a left carotid to subclavian artery bypass or subclavian artery transposition.^2^ After the public release of the thoracic branch endoprosthesis (TBE, W. L. Gore & Associates, Flagstaff, AZ), an on-label alternative to open LSA revascularization became available, facilitating proximal seal in zone 2 while maintaining perfusion to the left arm and the left vertebral artery via the retrograde side branch.^3^

Instead of a supraclavicular incision, the TBE only requires left upper extremity (LUE) access with instructions for use (IFU) recommending brachial access with axillary access as an alternative should the brachial artery be inadequate.^4^ Open axillary artery access is well-tolerated for large bore access, and the recent literature demonstrates percutaneous that axillary access is also a safe and feasible approach.^5^ Similarly, brachial artery access can be accomplished using either open or endovascular techniques; however, percutaneous brachial access has the additional risk of brachial sheath hematoma, which can require open repair for median nerve decompression to prevent permanent extremity dysfunction.^6^

The radial artery, although not within the IFU, is a theoretically safer access option because it is superficial and easily compressible. Percutaneous radial access is used frequently by interventional cardiologists with low reported complication rates.^7^ Given its reported success and safety in cardiology, it was hypothesized that similar percutaneous access techniques could be used for subclavian branch treatment with a TBE. The objective of this investigation was to characterize the safety profile of percutaneous radial access with the TBE and evaluate its effectiveness for subclavian branch treatment.

## Methods

A retrospective review of 63 consecutive patients who underwent TBE repair for zone 2 aortic pathology between 2022 and 2024 at a single academic hospital was performed. TBE repair indications included de novo aneurysmal disease, incorporating both isolated thoracic aneurysms and for creation of a proximal landing zone as a staged repair for thoracoabdominal aortic aneurysms; blunt thoracic aortic injuries (BTAIs); acute complicated type B aortic dissections; and penetrating aortic ulcers. The primary outcome was technical success, defined as the ability to achieve through-and-through wire access with successful delivery of the TBE and subclavian branch stents. Secondary outcomes were access site complications, subclavian side branch patency, and major complication rates.

**Fig 1:**
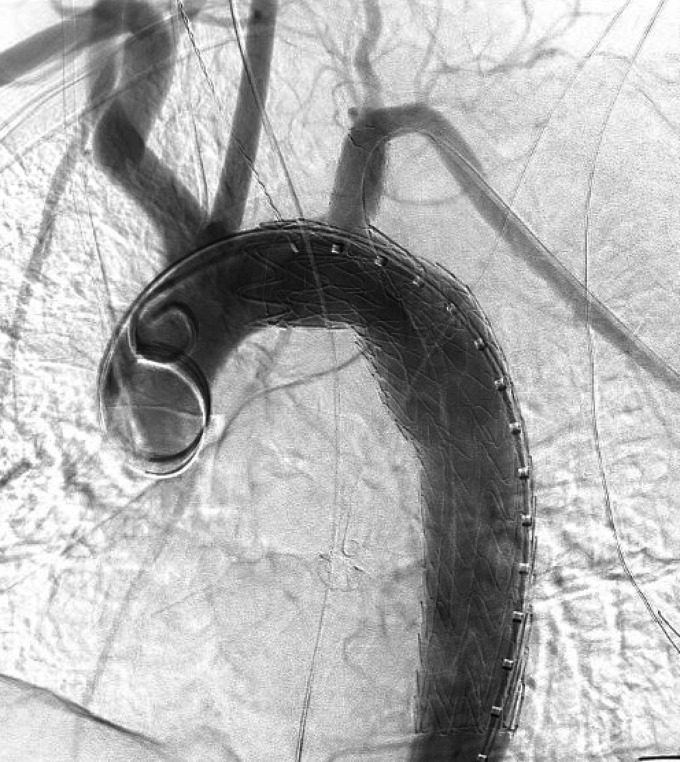
Completion aortic arch angiography demonstration exclusion of blunt thoracic aortic injury (BTAI) with patent left subclavian artery (LSA) side branch and antegrade filling of the left vertebral artery.

In terms of technical steps, after confirmation of a negative Allen's test and establishment of femoral access, left radial arterial access was performed under ultrasound guidance using the modified Seldinger technique resulting in placement of a 6F Slender sheath (Terumo Medical Corporation, Somerset, NJ) using the introducer kit. A radial cocktail consisting of 2 mg verapamil and 200 μg nitroglycerin was administered through the radial sheath. The patient was heparinized systemically, and a stiff angled Glidewire (Terumo Medical Corporation) was used in combination with a C2 catheter (Terumo Medical Corporation) to select the descending thoracic aorta. The sheath was then exchanged for a 6F radial-to-peripheral R2P sheath (Terumo Medical Corporation) of appropriate length. The rest of the procedure and delivery of the TBE device were performed in line with the manufacturer’s recommendations. Completion arch aortography was performed in all patients before sheath removal (Fig 1). Radial access site hemostasis was obtained using a TransRadial band (Terumo Medical Corporation). Side branch patency was evaluated with a computed tomography angiogram before discharge in patients with no contraindication to contrast and at the time of follow-up in patients with no contraindications who returned for follow-up.

## Results

**Fig 2:**
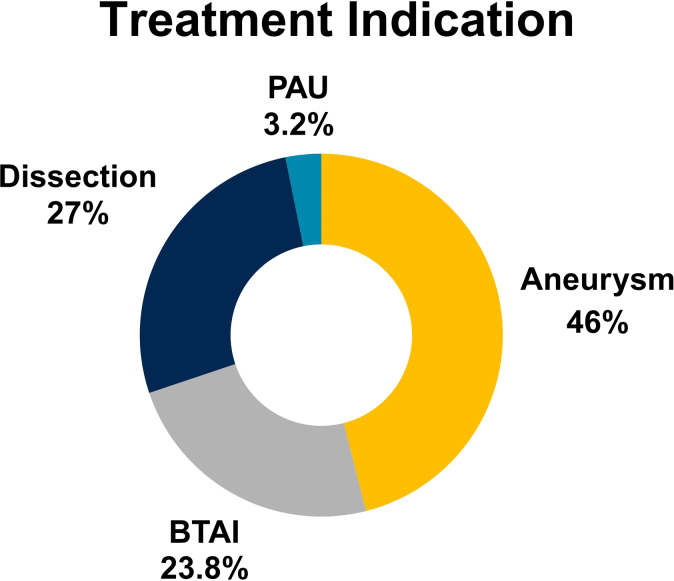
Distribution of treatment indications. *PAU*, penetrating aortic ulcer.

A total of 63 consecutive patients who underwent TBE repair of zone 2 thoracic aortic pathology between 2022 and 2024 were identified. The mean age of the cohort was 61.0 ± 16.5 years and 42 patients (66.7%) were male. The mean body mass index was 28.7 ± 6.3. The majority of patients were tobacco users (58.7%) and had a past medical history of hypertension (73.0%). Additional demographic characteristics are provided in Table I. The most common indication for repair was aneurysmal disease (n = 29 [46%]), followed by acute complicated aortic dissection (n = 17 [27%]), BTAI (n = 15 [23.8%]), and penetrating aortic ulcer (n = 2 [3.2%]) (Fig 2).

**Table I:** Demographics and medical comorbidities (n = 63)

Variables	Value
Age, years	61 ± 16.5
Male sex	42 (66.7)
BMI	28.7 ± 6.3
Tobacco use	37 (58.7)
Hypertension	46 (73.0)
Diabetes	6 (9.5)
COPD	13 (20.6)
Coronary artery disease	13 (20.6)
Hyperlipidemia	17 (27.0)
Prior stroke	9 (14.3)

*BMI,* Body mass index; *COPD,* chronic obstructive pulmonary disease.

Values are mean ± standard deviation or number (%).

Primary percutaneous radial access was attempted in 60 patients (95.2%), with the remaining 3 patients undergoing primary open brachial access secondary to either absence of the radial artery owing to previous coronary artery bypass grafting (n = 1) or inaccessibility from concomitant upper extremity trauma (n = 2) in the setting of BTAI. Technical success was achieved in 100% of radial access attempts with no instances of conversion to a more proximal arterial access site. In 60 radial accesses, only 1 access site complication (1.7%) was detected, an asymptomatic radial artery occlusion detected at follow-up that did not require intervention (Table II). The left vertebral artery and left subclavian side branch were evaluated on completion aortography, demonstrating 100% preservation and 100% patency rates, respectively, at case conclusion. Side branch patency was evaluated with a computed tomography angiogram in 58 patients (92.1%) before discharge. Forty-one patients (65.1%) returned for follow-up and more than 30 days; of these patients, 33 (52.4%) underwent computed tomography angiography confirming 100% LSA side branch patency.

**Table II:** Percutaneous radial access outcomes

Pathology	Percutaneous radial access attempted	Technical success of radial access	Radial access complications	Side branch patency of radial access
Aneurysmal disease (n = 29)	28 (96.5)	28 (100)	1 (1.7) asymptomatic thrombosis	27 (100)
BTAI (n = 15)	13 (86.7)	13 (100)	0	13 (100)
Aortic dissection (n = 17)	17 (100)	16 (100)	0	16 (100)
Penetrating aortic ulcer (n = 2)	2 (100)	2 (100)	0	2 (100)

*BTAI,* Blunt thoracic aortic injury.

Values are number (%).

There was no 30-day mortality. There was one instance of a type 2 non-ST elevation myocardial infarction (1.6%) and one instance of stroke in a patient undergoing repair of an acute complicated aortic dissection with an entry tear in zone 2 of the aorta. There was one case of respiratory failure/prolonged intubation and one instance of more than 1 L blood loss in the setting of an external iliac artery rupture secondary to large-bore sheath access (Table III).

**Table III:** Major complications

Complications	No. (%)
Death	0 (0)
Myocardial infarction	1 (1.6), type 2 NSTEMI
EBL > 1 L	1 (1.6), iliac rupture
Stroke/TIA	1 (1.6)
Respiratory failure/prolonged intubation	1 (1.6)

*EBL,* Estimated blood loss; *NSTEMI,* non-ST segment elevation myocardial infarction; *TIA,* transient ischemic attack.

## Discussion

In the elective setting, the Society for Vascular Surgery Guidelines for the management of the LSA recommend routine preoperative revascularization if the proximal seal requires coverage of the LSA. Traditionally, this goal is accomplished using a left carotid to subclavian bypass with concomitant TEVAR. Subclavian revascularization requires additional time under anesthesia or a separate procedure under general anesthesia with associated risks. In a study that included 112 patients who underwent concomitant carotid-subclavian bypass, a complication rate of 29% was reported. There were 27 instances of phrenic nerve palsy, 6 instances of recurrent laryngeal nerve palsy, and 1 instance of neck hematoma requiring reexploration. Two patients developed a late anastomotic pseudoaneurysm that required intervention. A 30-day mortality rate of 5% was also reported. The 5-year patency rate was excellent at 97%.^1^ Although a durable repair, a carotid-subclavian bypass has a risk of complications that may be under-reported in the literature and highlights an area for patient outcome improvement. The now commercially available TBE circumvents the need for left carotid-subclavian bypass by maintaining LUE and left vertebral perfusion via its left subclavian side branch, decreasing the risks associated with open revascularization. It is also performed more efficiently with a median operative time of 93 minutes in our experience when performed as an isolated procedure.

The TBE IFU recommend left brachial artery access with an alternative of more proximal access should the brachial artery not be adequate. In the initial investigational device exemption data collection period, 68.5% of LUE accesses were obtained in the brachial artery, 4.6% in the axillary artery, and 25.6% in other arteries, which, per the IFU “may include a combination of multiple access sites including radial access.”^4^ Given the high reported rates of percutaneous brachial access complications (7.5%) and open brachial access requiring an additional incision with added anesthesia time (reported complication rate of 1.6%), we trialed the off-IFU use of percutaneous left radial artery for LUE through-and-through wire access given its proven success in cardiology.^6^^,^^7^

Our overall access site complication rate of 1.7% is lower than that reported in the cardiology and vascular surgery literature, although our sample size is relatively small.^7^^,^^8^ A pooled analysis of several cardiology randomized controlled trials consisting of more than 3500 patients suggests that radial occlusion, the most common complication of radial access, occurs in approximately 3.0% of similarly accessed patients (5F sheath, intra-arterial nitroglycerin, verapamil, and heparin at cannulation). They also reported a risk of major bleeding and/or large hematomas in 1.4% of cases, which was not seen in our cohort. This difference could be explained potentially by this study's comparatively small sample size and diligent use of the TransRadial band (Terumo Medical Corporation) in a protocolized fashion. Alternatively, the counterpuncture technique (where the needle is advanced through both walls of the artery and subsequently withdrawn into the lumen) used in some of the included cardiology trials may place patients at a higher risk for complications than the direct ultrasound-guided intraluminal access performed in this study.^7^

In a recent Vascular Quality Initiative study that compared the safety of radial, brachial, and femoral access for peripheral arterial disease intervention, an overall radial access complication rate of 2.2% across 270 accesses was reported. The most common complications in this cohort were hematoma formation in 1.5% of cases and radial pseudoaneurysm development in 0.4% of cases. Although the overall complication rate reported in the Vascular Quality Initiative study is more comparable with our data, there were no instances of radial access site occlusion. This finding potentially reflects limitations of registry data, which may not identify long-term access site complications that are discovered outside of the index procedure and index hospitalization, as the instance of radial occlusion was in this cohort from a retrospective review of clinic follow-up physical examination documentation.^8^

There were several major complications in our cohort, albeit at a lower rate than standard TEVAR. There was one instance (1.6%) of major blood loss (>1 L) in the setting of an external iliac artery rupture. This complication was secondary to the larger sheath size required for delivery of the TBE device. One stroke occurred in this cohort during treatment of an acute complicated type B dissection that originated in zone 2 with renal malperfusion. Treatment of acute complicated type B aortic dissections has a published stroke rate of 6.8% in STABLE II (Use of the Zenith® Dissection Endovascular System in the Treatment of Patients With Acute, Complicated Type B Aortic Dissection).^9^ Although the subgroup of acute complicated type B dissections treated with the TBE using radial access was too small in this study for a meaningful subgroup analysis, this complication highlights the importance of avoiding treatment of aortic dissections during the acute phase unless necessary.

Overall, this study demonstrates that left radial arterial offers a safe and reasonable access site for subclavian branch treatment during TBE repair. The access site complication rate was 1.7%, with the only complication being an asymptomatic radial occlusion, the most common complication of radial access.^7^ The rate of major complications encountered is likely similar to on-IFU brachial or axillary access, given that these complications were seemingly unrelated to the radial artery, but rather the risks traditionally associated with TEVAR and large-bore sheath access. A larger cohort, potentially with multicenter involvement, is needed to confirm these findings; however, the initial results of radial access suggest it may be a safe and equally effective technique compared with more proximal arterial access sites.

There are some limitations to this study. Data were gathered in a retrospective manner from a single institution with the majority of cases performed by an experienced TBE user. The data reported in this study may not be generalizable to low-volume, less experienced surgeons and sites; however, provided the operator is experienced with radial access, it should not lead to any increased risk compared with on-IFU access. The reported access site complication rate may also be slightly lower than true values, given that the most common complication of radial access is asymptomatic occlusion, which may not be detected in many cases because there was no dedicated radial artery imaging performed at follow-up, relying only on physical examination. Some patients did not return for follow-up, had contraindications to additional contrast administration, or had received all necessary imaging before discharge, explaining the limited number of patients who have had imaging after 30 days at the time of data collection.

## Conclusions

Left radial access is a safe technique for establishing the through-and-through wire access necessary for the treatment of the left subclavian branch during TBE with an access site complication rate of only 1.7%, a 100% technical success rate, and a 100% side-branch patency rate. Although a larger, multicenter experience is necessary to confirm these findings, radial access appears to be a safe and equally effective alternative for branch treatment during TBE.

## Funding

None.

## Disclosures

S.M. is a consultant for W. L. Gore & Associates.
